# Refractory Ventricular Fibrillation Successfully Managed with Thrombolysis and Prolonged Resuscitation: A Case Report

**DOI:** 10.7759/cureus.94763

**Published:** 2025-10-17

**Authors:** Zakia R Aslam, Ahmed Ghoniem

**Affiliations:** 1 Emergency Medicine, Queens Hospital, London, GBR; 2 Emergency, King George Hospital, London, GBR

**Keywords:** cardiac arrest, case report, mechanical cpr, refractory ventricular fibrillation, thrombolysis

## Abstract

Refractory ventricular fibrillation (VF) is a critical condition that often presents with a poor prognosis and significant resuscitation challenges. We report the case of a 43-year-old man presenting with acute chest pain who sustained recurrent VF cardiac arrest in the emergency department. Due to prolonged resuscitation involving 21 defibrillation shocks, repeated advanced life support, and administration of thrombolysis, return of spontaneous circulation (ROSC) was achieved. Subsequent coronary angiography revealed severe in-stent restenosis in the left anterior descending artery, with successful percutaneous coronary intervention. This case highlights the importance of early recognition of refractory VF, the potential role of adjunctive therapies such as thrombolysis, and the value of multidisciplinary collaboration in achieving favourable outcomes.

## Introduction

Refractory ventricular fibrillation (VF) is defined as persistent VF despite at least three defibrillation attempts, 300 mg of amiodarone, and 3 mg of adrenaline [1,2]. It represents one of the most severe forms of cardiac arrest, and survival rates in this context are extremely poor, with historical data suggesting mortality approaching 97% [1]. Management typically includes high-quality cardiopulmonary resuscitation (CPR), defibrillation, and pharmacological support. For patients who do not respond to these standard measures, more advanced strategies - such as double sequential external defibrillation, vector-change shocks, beta-blockade, or extracorporeal CPR - have been explored to improve outcomes [1,2]. Understanding these options is important because timely recognition and escalation of care can make a critical difference. Here, we present a case of in-hospital refractory VF managed with prolonged advanced life support, thrombolysis, and subsequent revascularisation, which resulted in a favourable neurological outcome.

## Case presentation

**Figure 1:**
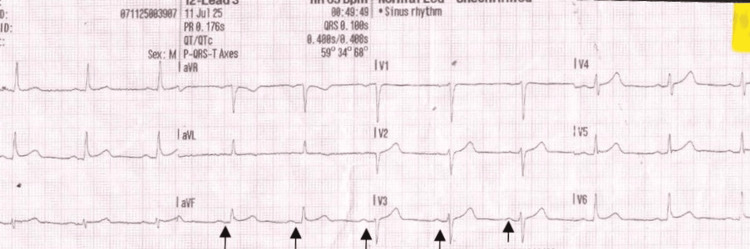
Initial ECG on arrival showing sinus rhythm without ST-segment deviation. Initial ECG on arrival demonstrating sinus rhythm without acute ST-segment deviation or dynamic ischemic changes. The absence of clear ST elevation or depression at presentation suggested no immediate evidence of ongoing transmural ischemia, although subtle anterior attenuation of R-wave amplitude was noted and monitored with serial ECGs.

A 43-year-old man presented to the emergency department (ED) at 01:25 hrs with sudden-onset, sharp, crushing chest pain following sexual activity. His past medical history included myocardial infarction in 2016 (treated with two stents), type 2 diabetes, hypercholesterolaemia, ulcerative colitis, and hypertension. He was an ex-smoker and had recently started on inclisiran, a small interfering RNA (siRNA)-based PCSK9 synthesis inhibitor (June 2025). There was no family history of premature coronary artery disease. On arrival, he was clammy and diaphoretic but alert, with stable vital signs (respiratory rate (RR) 18 breaths per minute, heart rate (HR) 71 bpm, blood pressure (BP) 107/68 mmHg, oxygen saturation (SpO₂) 99%). The initial ECG demonstrated sinus rhythm without acute ST-segment changes, indicating no immediate evidence of transmural ischemia at presentation (Figure 1).

**Figure 2:**
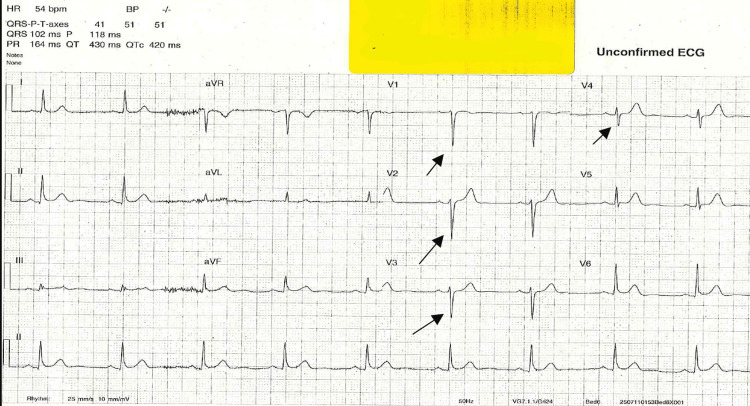
ECG showing sinus bradycardia with poor R wave progression ECG demonstrating sinus bradycardia (heart rate ~54 bpm) with poor R-wave progression across the anterior precordial leads (V1–V4). This pattern may indicate evolving anterior ischemia or conduction delay, consistent with dynamic myocardial changes observed during the patient’s deterioration.

His chest pain partially improved with glyceryl trinitrate and morphine. The working diagnosis was acute coronary syndrome, and management included continuous cardiac monitoring, serial cardiac enzymes, and chest radiography. At 01:53 hrs, a repeat ECG A repeat ECG demonstrated sinus bradycardia (54 bpm) with poor R-wave progression in the anterior leads, suggestive of evolving ischemic changes (Figure 2).

Chest pain recurred at 03:00 hrs without new ECG findings. At 03:35 hrs, he suddenly collapsed with agonal respirations and was found pulseless in ventricular fibrillation (VF). Advanced life support was initiated immediately, including high-quality CPR, early defibrillation, adrenaline, and amiodarone [1,2]. A mechanical CPR device (LUCAS, Jolife AB, Lund, Sweden) was deployed to maintain consistent compressions. Despite multiple defibrillation attempts, the patient remained in refractory VF.

As he was not eligible for immediate percutaneous coronary intervention (PCI) [3], systemic thrombolysis with alteplase was administered (50 mg at 03:48 hrs and a further 50 mg at 04:02 hrs) [4-6]. Resuscitation efforts continued for approximately 55 minutes, during which 21 defibrillation shocks, repeated doses of adrenaline (1 mg every three to five minutes), and a total of 600 mg of amiodarone were given. Return of spontaneous circulation (ROSC) was achieved at 04:25 hrs, with new ST-elevation observed post-ROSC. Table 1 summarises the deterioration and arrest timeline.

**Table 1:** Deterioration and Arrest Timeline VF: Ventricular fibrillation; NSR: normal sinus rhythm; CPR: cardiopulmonary resuscitation; ROSC: return of spontaneous circulation.

Time (hrs)	Event
03:00	Chest pain recurred; monitor NSR
03:35	Collapse → VF arrest; CPR and shocks started
03:48	Alteplase 50 mg administered
04:02	Second 50 mg alteplase given
04:25	ROSC achieved; ST-elevation noted

Following ROSC, the patient was intubated and mechanically ventilated in volume control/SIMV mode (fraction of inspired oxygen (FiO₂) ~0.60). Sedation with propofol and remifentanil was initiated for comfort and to control agitation. The LUCAS device was removed once spontaneous circulation was stable. Invasive monitoring was established via arterial and central venous lines. Arterial blood gas analysis revealed severe metabolic acidosis (pH ~6.7, lactate ~18 mmol/L). Sodium bicarbonate and intravenous fluids were administered to correct acidosis and optimise perfusion, with serial blood gases showing gradual lactate improvement as haemodynamics stabilised. Table 2 summarises post-resuscitation metabolic and ventilatory parameters.

**Table 2:** Arterial blood gas of patient with metabolic acidosis "-" represents not recorded or not available values.

Parameter	Result	Unit	Reference Range
pH	-	-	7.35-7.45
Partial pressure of carbon dioxide (pCO_2_)	6.66	kPa	4.30-6.10
Partial pressure of oxygen (pO_2_)	26.1	kPa	9.50-13.9
Total haemoglobin	135	g/L	123-175
Oxygen saturation	97.1	%	94-98
Oxyhaemoglobin	95.6	%	90-95
Carboxyhaemoglobin	0.1	%	0.0-2.2
Deoxyhaemoglobin	2.9	%	-
Methemoglobin	1.4	%	0.0-1.2
Potassium	3.4	mmol/L	3.5-5.3
Sodium	143	mmol/L	133-146
Ionised calcium	1.05	mmol/L	1.17-1.32
Chloride	-	mmol/L	95-105
Glucose	20.6	mmol/L	3.8-5.6
Lactate	18	mmol/L	0.0-1.3
Creatinine	-	µmol/L	53-106
Urea	-	mmol/L	2.5-6.4
Partial pressure of carbon dioxide (temperature-corrected)	6.66	kPa	4.7-6.0
Partial pressure of oxygen (temperature-corrected)	26.1	kPa	10.6-13.3
Oxygen content	18.5	Vol%	16-20

**Figure 3:**
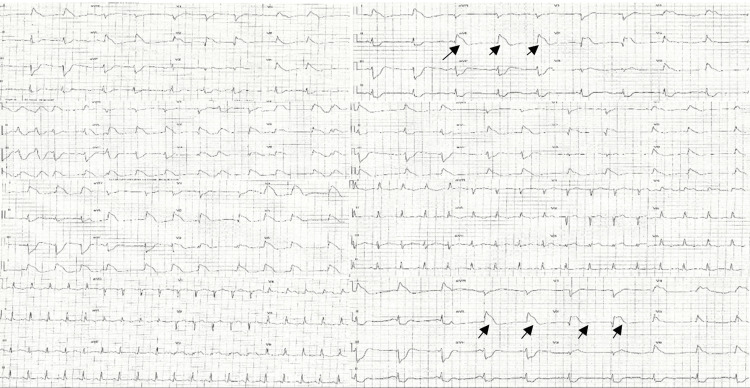
Post-ROSC ECGs taken at different intervals. Post-ROSC ECGs obtained at sequential intervals demonstrating a classic “shark-fin” or “lambda-wave” pattern, characterised by fusion of the QRS complex, ST segment, and T wave into a broad, triangular morphology (black arrows). This finding is most consistent with severe transmural ischemia, typically associated with proximal left anterior descending (LAD) coronary artery occlusion. The evolution of this waveform post-resuscitation reflects ongoing myocardial injury prior to reperfusion. ROSC: Return of spontaneous circulation.

Sequential ECGs obtained after ROSC demonstrated the characteristic shark-fin (lambda-wave) morphology - a fusion of the QRS, ST, and T waves - highly suggestive of ongoing transmural ischemia due to proximal left anterior descending (LAD) occlusion (Figure 3).

Once stabilised, the patient was transferred to a tertiary centre for coronary angiography, which revealed severe in-stent restenosis in the proximal/mid left anterior descending artery and diffuse disease in the left circumflex artery. Successful PCI was performed [7].

The post-PCI course was notable for recovery from acute kidney injury, preservation of left ventricular ejection fraction (~50%), and absence of neurological deficit. The patient was extubated, mobilised, and enrolled in structured cardiac rehabilitation. He was discharged in late July on dual anti-platelet therapy and secondary prevention medications, with follow-up at the acute myocardial infarction and lipid clinics. 

## Discussion

This case illustrates the successful management of refractory VF through a combination of prolonged advanced life support, mechanical CPR, and thrombolysis, culminating in revascularisation and good neurological recovery. Refractory VF is a rare but devastating event with historically poor survival [1,2]. Recognition of refractory VF should prompt consideration of advanced strategies beyond guideline-based CPR, including double sequential external defibrillation, vector change shocks, beta-blockade, and extracorporeal CPR [1,2].

The use of thrombolysis in this case warrants particular discussion. While not routinely recommended for refractory VF of presumed myocardial infarction origin, its administration was guided by the strong suspicion of an underlying coronary thrombotic occlusion and the unavailability of immediate PCI. Large randomised trials, such as Thrombolysis in Cardiac Arrest (TROICA), have not shown a mortality benefit with routine thrombolysis during cardiac arrest [5]; however, physiological reasoning supports its potential role in select patients where coronary thrombosis is the likely precipitant and mechanical reperfusion cannot be achieved promptly [5,6]. In this case, the patient’s eventual ROSC following administration of alteplase suggests that thrombolysis may have contributed to reperfusion of the occluded vessel, enabling successful defibrillation and restoration of spontaneous circulation. The subsequent angiographic findings of in-stent restenosis support this interpretation.

Additionally, the patient’s prolonged resuscitation course demonstrates the physiological capacity for neurological recovery even after extended low-flow states when high-quality mechanical CPR is maintained. The gradual correction of metabolic acidosis and improvement in lactate levels post-ROSC further indicate effective perfusion and metabolic recovery.

Overall, this case highlights the importance of dynamic, multidisciplinary decision-making during cardiac arrest management. It underscores that even in prolonged arrests with refractory VF, favourable outcomes are possible through sustained efforts, mechanical support, and thoughtful integration of adjunctive therapies, including, in carefully selected cases, the considered use of thrombolysis [8-12].

## Conclusions

Refractory VF carries an extremely poor prognosis, yet this case demonstrates that favourable outcomes may be achievable with early recognition, coordinated multidisciplinary efforts, and the considered use of adjunctive therapies such as thrombolysis. In this instance, thrombolysis was employed as a last-resort measure in the absence of immediate PCI availability and may have contributed to successful ROSC; however, causality cannot be firmly established. The case underscores the importance of identifying reversible causes during ongoing resuscitation and being prepared to escalate beyond conventional algorithms when clinically justified. While this report supports further exploration of advanced resuscitation strategies - including thrombolysis, mechanical circulatory support, and tailored post-arrest care - it also highlights the need for cautious interpretation, given the single-patient nature of the evidence and the broader uncertainty surrounding thrombolysis efficacy in refractory VF.
